# Association between HDL-C levels and menopause: a meta-analysis

**DOI:** 10.1007/s42000-020-00216-8

**Published:** 2020-06-18

**Authors:** Hongwei Li, Runlu Sun, Qian Chen, Qi Guo, Junjie Wang, Liming Lu, Yuling Zhang

**Affiliations:** 1 Cardiovascular Medicine Department, Sun Yat-sen Memorial Hospital, Sun Yat-sen University, No. 107, the West of Yanjiang Road, Guangzhou, 510120 China; 2 Guangdong Province Key Laboratory of Arrhythmia and Electrophysiology, Guangzhou, 510120 China; 3 Clinical Research Center, South China Research Center for Acupuncture and Moxibustion, Medical College of Acu-Moxi and Rehabilitation, Guangzhou University of Chinese Medicine, Guangzhou, 510006 China

**Keywords:** High-density lipoprotein-cholesterol, Low-density lipoprotein-cholesterol, Triglycerides, Total cholesterol, Postmenopausal, Premenopausal, Cross-sectional

## Abstract

**Purpose:**

Menopause modifies women’s lipid profiles. However, the fact that it is still unclear whether high-density lipoprotein-cholesterol (HDL-C) levels decrease in postmenopausal women necessitated a systematic review and meta-analysis.

**Methods:**

The PubMed, EMBASE, Cochrane Library, and Web of Science databases were searched and 498 articles published between 1987 and 2020 were retrieved. Studies reporting HDL-C, low-density lipoprotein-cholesterol (LDL-C), total cholesterol (TC), and triglyceride (TG) levels in both postmenopausal and premenopausal populations were included. The quality of the included studies was assessed using the Cross-Sectional/Prevalence Study Quality tool. The standard mean difference (SMD) and 95% confidence interval (CI) were estimated using random effects models. A meta-regression analysis and subgroup analysis were performed to identify potential modifiers. Egger’s test and funnel plots were constructed to evaluate publication biases.

**Results:**

Lipid profiles from 18 cross-sectional studies and two cohort studies including 5652 postmenopausal women and 7825 premenopausal women were meta-analyzed. HDL-C levels were not significantly different between the postmenopausal and premenopausal women (SMD = − 0.053, 95% CI − 0.171 to 0.066, *p* = 0.383) and were not affected by country, publication year, study quality in the meta-regression analysis, or significant publication bias. Higher LDL-C, TC, and TG levels were detected in postmenopausal women than in premenopausal controls.

**Conclusion:**

Unlike increased LDL-C, TC, and TG levels, HDL-C levels in pre- and postmenopausal women were not different in this first meta-analysis of lipid profiles in premenopausal and postmenopausal women. Prospective studies with large populations examining HDL-C levels and functions in women with different menopausal statuses are essential in the future.

**Trial registration number:**

None.

**Electronic supplementary material:**

The online version of this article (10.1007/s42000-020-00216-8) contains supplementary material, which is available to authorized users.

## Introduction

The menopause transition is an inevitable physiological process that occurs in women and is characterized by biological changes such as reductions in estradiol levels and proatherogenic lipid profiles [1]. An increased risk of cardiovascular disease (CVD) has been observed during this phase of life. In particular, the risk doubles in women aged 45–54 years [2], indicating harmful changes in the metabolic pattern between the pre- and postmenopausal periods. High-density lipoprotein cholesterol (HDL-C), a robust, independent, and traditional CVD risk factor, has exhibited inconsistent changes in a quantitative analysis of menopause-related changes. In addition to the dichotomous findings of either lower or higher HDL-C levels in postmenopausal women [3–5], recent cross-sectional studies did not identify a significant difference in HDL-C levels between pre- and postmenopausal women [6, 7]. The long-established view acknowledges that HDL-C levels negatively correlate with the risk of CVD in the overall population [8]. However, accumulating evidence of a positive association between HDL-C levels and atherosclerosis-related diseases in postmenopausal women challenges the cardioprotective effect of high HDL-C levels [9, 10]. An open-ended question is whether a high HDL-C level is a CVD risk marker after menopause. Elevated levels of low-density lipoprotein cholesterol (LDL-C), triglyceride (TG), and total cholesterol (TC) are related to CVD, with substantial interrelationships with HDL-C levels. An improved understanding of the changes in the levels of HDL-C and other lipids in response to menopause status will ensure a more reliable investment in strategies aiming to reduce CVD risk.

To our knowledge, we have performed the first meta-analysis to clarify whether HDL-C, LDL-C, TG, and TC levels are altered after the menopausal transition. The results of the meta-regression and subgroup analyses may explain the heterogeneity of changes in HDL-C levels during the menopause transition.

## Methods

### Data sources and database searches

We searched four public electronic databases, namely PubMed, Web of Science, EMBASE, and the Cochrane Library, for related articles. The following search terms were used: “Postmenopause” or “Postmenopausal period,” and “Premenopause” or “Premenopausal period” in combination with “Lipoproteins, HDL” or “High-density Lipoproteins.” The search was restricted to articles published in the English language that included human participants. Subsequent to our search, the articles included studies published from the beginning of 1987 to 2020. All titles and abstracts yielded by the search were then screened for potential suitability. Those articles that appeared to be relevant were retrieved and examined in more detail. This search was supplemented by manual searches of the reference lists of all retrieved studies. We followed the Meta-analysis of Observational Studies in Epidemiology (MOOSE) guidelines to conduct and report the results of our meta-analyses [11].

### Inclusion and exclusion criteria

We included comparative studies of postmenopausal and premenopausal women published in the English language that met the following criteria: (1) original articles that included, (2) both healthy pre- and postmenopausal women, and (3) simultaneously reported HDL-C, LDL-C, TC, and TG levels. Postmenopausal status was defined as more than 12 months of amenorrhea and premenopausal state was defined as regular menstrual cycles. Studies were excluded when (1) the pre- or postmenopausal women had lipid-altering diseases, including diabetes, coronary artery disease, malignancy, and thyroid functional abnormalities; (2) the pre- or postmenopausal women received hormone replacement therapy (HRT) or other medications affecting lipid metabolism; (3) the premenopausal women were pregnant; or (4) the women underwent menopause due to surgery or other unnatural causes. Studies were also excluded if they used only menopause status as a covariate or if they reported overlapping data or consisted of studies of a single case or reviews.

### Data extraction and methodological quality assessment

Two authors (HWL and QC) independently extracted and summarized the data from the included studies. The papers were required to have indicated the total numbers of postmenopausal subjects and premenopausal subjects as well as the mean HDL-C level ± standard deviation (SD) or the mean HDL-C level ± standard error of mean (SE). When multiple publications reported the same or overlapping data, we included the most recent study with the largest sample size. The following data were extracted from each study: first author, year, country, sample sizes of the case and control groups, mean age, and the levels of TC, TG, LDL-C, and HDL-C, as well as their SD or SE. We extracted the baseline data from cohort studies if multiple time points were provided. The level of quality of all cross-sectional studies was evaluated using the Cross-Sectional/Prevalence Study Quality tool recommended by the Agency for Healthcare Research and Quality. An item received a score of “1” if the answer was “Yes” and a score of “0” if the answer was “No” or “Unclear” [12]. Studies with scores of 0–3 points were classified as “low quality,” studies with a score of 4–7 points were classified as “moderate quality,” and studies with a score of 8–11 points were classified as “high quality” [13]. Two reports were cohort studies [5, 14], and the same checklist was also used to assess their quality. One of the selected papers was a high-quality study and the remaining 19 papers were moderate quality. The majority of the included studies were considered to display an acceptable level of quality [see Table S1 in Additional file 1].

### Statistical analysis

All statistical analyses were conducted using STATA software (version 15.0). A two-tailed *p* value of < 0.05 for any test or model was considered significant. All extracted data on TC, HDL-C, LDL-C, and TG levels were converted into mg/dL. The pooled standardized mean difference (SMD) and its 95% confidence interval (CI) were calculated for the meta-analysis of the lipid (HDL-C, LDL-C, TC, and TG) levels, which were included as continuous variables, in both cross-sectional studies and the baseline data from cohort studies, as mentioned in similar studies [15]. Heterogeneity was assessed using the *I*^2^ statistic, which describes the percentage of variability in the effect estimates due to heterogeneity rather than chance. An approximated normality test (*z*-test) was used to analyze the aggregated results. Heterogeneity was considered significant when the probability value of the chi-square test was < 0.05. As recommended by *the Cochrane Handbook for the Systematic Reviews of Interventions*, an *I*^2^ value < 40% suggests that “heterogeneity might not be important,” whereas an *I*^2^ value > 75% suggests “considerable heterogeneity.” We pooled the data across trials using the fixed effects model (Mantel-Haenszel) if heterogeneity was absent or the random effects model if heterogeneity was present. A meta-regression was performed to explore the effects of publication year, sample size, country, and study quality on the effect estimates and identified the potential sources of the heterogeneity. Subgroup analyses were performed after stratification according to the publication year, sample size, and country to evaluate the association between HDL-C levels and menopausal status. Additionally, a sensitivity analysis was performed by repeating the meta-analysis after one study or a few studies were sequentially removed to assess whether a particular study significantly affected the pooled estimates. Potential publication bias was tested by creating a funnel plot that represented the standard error as the measure of the sample size and SMD as the measure of the pooled effect and Egger’s test.

## Results

### Search results

**Fig. 1:**
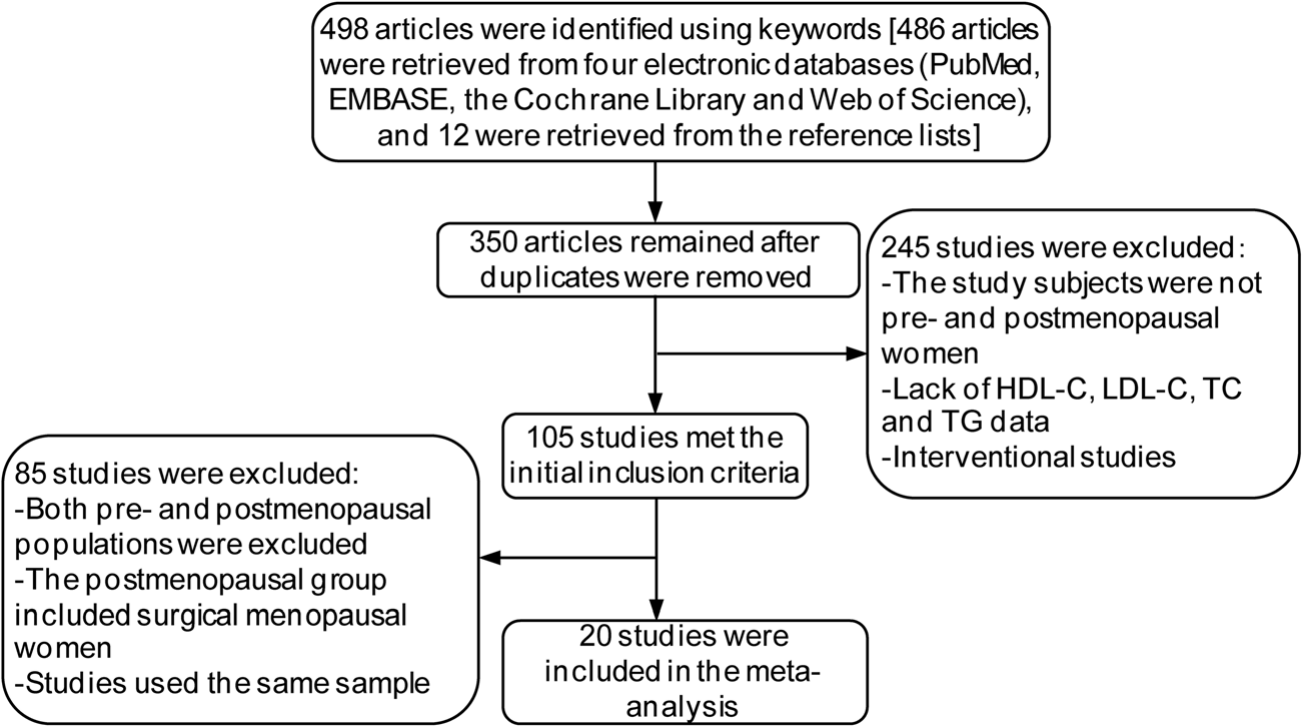
Flow chart of the literature search and study selection process

The literature search yielded 498 related papers. Three hundred fifty studies remained after removing duplicates. Two hundred forty-five articles were then excluded because they did not include pre- and postmenopausal women as subjects; lacked HDL-C, LDL-C, TC, and TG data; and were interventional studies. One hundred and five studies met the initial inclusion criteria, of which 85 studies were excluded because of exclusion of both pre- and postmenopausal populations, inclusion of surgical menopausal women, and studies using the same populations. The remaining 20 original articles were included in the meta-analysis. The literature selection criteria and the reasons for exclusion are presented in Fig. 1. Eighteen of the 20 original articles were cross-sectional studies, and the remaining two articles were cohort studies.

### Study characteristics

The characteristics of all 20 selected studies, which included 5652 postmenopausal women and 7825 premenopausal women, are summarized in Table 1 [4, 5, 7, 14, 16–31]. Asian participants from China, Korea, Japan, and India included 4979 postmenopausal women (88.1%) and 7132 premenopausal controls (91.1%), while non-Asian participants consisted of 673 postmenopausal women (11.9%) and 693 premenopausal females (8.9%). These eligible studies were conducted in ten countries, four of which were from the USA [4, 14, 23, 27], three articles were from Argentina [18, 24, 25], three articles were from Korea [19, 20, 28], two articles were from Japan [22, 31], two articles were from China [5, 23], and two articles were from Slovakia [7, 17]. The other four articles were from Brazil [21], India [16], Turkey [30], and the Netherlands [29].

**Table 1:** Characteristics of the 20 included studies

First author	Year	Study design	Country	Number	Age, years	HDL-C (mg/dl)	LDL-C (mg/dl)	TG (md/dl)	TC (mg/dl)
Cernanova et al. [7]	2016	Cross-sectional	Slovakia	Postmenopausal women: 146 Premenopausal women: 146	53.51 ± 4.52 45.73 ± 3.77	61.16 ± 16.26 62.32 ± 16.26	126.76 ± 41.74 120.97 ± 32.85	127.43 ± 85.84 110.62 ± 60.18	211.97 ± 43.63 204.25 ± 35.91
Mogarekar and Kulkarni [16]	2015	Cross-sectional	India	Postmenopausal women: 40 Premenopausal women: 40	59.10 ± 10.04 30.13 ± 7.20	30.93 ± 7.47 36.68 ± 7.95	148.55 ± 40.54 135.13 ± 34.52	167.53 ± 68.79 118.95 ± 43.41	212.38 ± 48.43 195.35 ± 36.62
Luptakova et al. [17]	2012	Cross-sectional	Slovakia	Postmenopausal women: 129 Premenopausal women: 167	53.30 ± 0.32 45.59 ± 0.26	58.06 ± 17.41 61.16 ± 15.10	128.70 ± 39.42 123.29 ± 34.78	139.82 ± 93.81 115.93 ± 59.29	214.29 ± 40.93 206.56 ± 36.29
Muzzio et al. [18]	2011	Cross-sectional	Argentina	Postmenopausal women: 23 Premenopausal women: 13	53.00 ± 4.25 33.00 ± 5.25	52.26 ± 12.00 60.39 ± 13.16	168.89 ± 48.31 109.37 ± 31.30	138.05 ± 64.60 69.03 ± 36.28	241.70 ± 47.88 187.64 ± 31.27
Jeon et al. [19]	2011	Cross-sectional	Korea	Postmenopausal women: 805 Premenopausal women: 1166	51.20 ± 9.00 49.30 ± 8.50	61.60 ± 13.90 61.50 ± 13.20	130.60 ± 31.10 109.90 ± 27.40	95.00 ± 41.90 83.20 ± 37.40	209.40 ± 15.40 186.00 ± 30.80
Zhou et al. [5]	2010	Cohort	China	Postmenopausal women: 349 Premenopausal women: 380	53.80 ± 2.80 42.20 ± 3.80	60.20 ± 14.20 58.70 ± 12.30	117.40 ± 32.00 103.70 ± 25.70	118.00 ± 3.44 90.00 ± 2.37	204.50 ± 35.90 182.80 ± 28.60
Jeong et al. [20]	2010	Cross-sectional	Korea	Postmenopausal women: 2661 Premenopausal women: 4613	58.60 ± 6.40 43.50 ± 5.90	57.40 ± 13.60 60.00 ± 13.60	133.10 ± 31.00 112.80 ± 28.00	109.00 ± 66.20 82.40 ± 48.50	212.20 ± 33.50 189.30 ± 31.80
Giribela et al. [21]	2009	Cross-sectional	Brazil	Postmenopausal women: 18 Premenopausal women: 22	48.00 ± 3.00 45.00 ± 3.00	61.00 ± 14.00 61.00 ± 12.00	100.00 ± 24.00 103.00 ± 35.00	80.00 ± 44.00 103.00 ± 50.00	178.00 ± 24.00 191.00 ± 27.00
Karita et al. [22]	2008	Cross-sectional	Japan	Postmenopausal women: 59 Premenopausal women: 68	54.90 ± 3.40 42.00 ± 4.70	70.50 ± 12.50 71.40 ± 12.60	144.00 ± 37.00 127.00 ± 32.00	90.60 ± 48.40 65.80 ± 23.40	226.00 ± 38.00 200.00 ± 28.00
Lin et al. [23]	2006	Cross-sectional	China	Postmenopausal women: 234 Premenopausal women: 360	53.10 ± 4.40 46.00 ± 3.60	61.94 ± 17.03 56.52 ± 14.71	131.01 ± 35.94 125.60 ± 30.14	116.81 ± 71.68 91.15 ± 59.29	143.63 ± 44.02 144.40 ± 40.93
Zern et al. [24]	2005	Cross-sectional	USA	Postmenopausal women: 20 Premenopausal women: 24	58.50 ± 7.50 39.70 ± 8.50	69.68 ± 13.55 65.81 ± 8.90	112.08 ± 33.62 112.08 ± 30.92	132.74 ± 106.19 88.50 ± 48.67	216.22 ± 37.84 193.05 ± 39.77
Zago et al. [25]	2004	Cross-sectional	Argentina	Postmenopausal women: 30 Premenopausal women: 28	52.90 ± 9.90 32.70 ± 9.90	52.00 ± 17.52 60.10 ± 19.04	167.10 ± 66.26 108.50 ± 44.93	137.00 ± 89.10 69.00 ± 51.79	244.30 ± 67.02 187.10 ± 44.93
Berg et al. [26]	2004	Cross-sectional	Argentina	Postmenopausal women: 30 Premenopausal women: 20	57.00 ± 5.30 36.90 ± 4.10	58.45 ± 13.16 58.45 ± 10.06	154.59 ± 44.06 109.37 ± 23.96	124.78 ± 44.25 72.57 ± 22.12	237.84 ± 47.10 186.87 ± 22.01
Kanaley et al. [27]	2001	Cross-sectional	USA	Postmenopausal women: 27 Premenopausal women: 23	53.30 ± 4.24 49.00 ± 2.83	57.40 ± 30.03 53.60 ± 22.08	120.20 ± 60.06 115.60 ± 50.34	102.10 ± 91.85 95.00 ± 90.08	193.90 ± 68.89 194.90 ± 79.49
Kim et al. [28]	2000	Cross-sectional	Korea	Postmenopausal women: 821 Premenopausal women: 485	47.20 ± 2.90 47.10 ± 2.60	207.40 ± 34.70 190.00 ± 33.90	70.00 ± 17.30 66.50 ± 16.80	123.60 ± 27.20 112.50 ± 25.40	118.30 ± 69.10 102.00 ± 60.70
Peters et al. [29]	1999	Cross-sectional	The Netherlands	Postmenopausal women: 93 Premenopausal women: 93	51.10 ± 2.20 50.60 ± 2.40	250.19 ± 52.51 227.41 ± 52.51	63.48 ± 21.68 61.16 ± 21.68	166.96 ± 47.54 146.09 ± 47.54	102.65 ± 72.57 102.65 ± 72.57
Oner et al. [30]	1997	Cross-sectional	Turkey	Postmenopausal women: 18 Premenopausal women: 20	57.80 ± 5.10 28.90 ± 3.70	204.00 ± 38.80 153.00 ± 29.10	44.20 ± 7.80 51.10 ± 8.00	137.00 ± 37.40 87.20 ± 25.70	113.00 ± 37.70 71.10 ± 21.30
Li et al. [4]	1996	Cross-sectional	USA	Postmenopausal women: 74 Premenopausal women: 72	55.80 ± 7.40 41.20 ± 6.50	225.10 ± 34.30 193.40 ± 32.60	56.40 ± 14.10 61.00 ± 15.70	144.90 ± 29.70 117.60 ± 29.00	117.90 ± 69.10 72.80 ± 35.00
Wakatsuki and Sagara [31]	1994	Cross-sectional	Japan	Postmenopausal women: 10 Premenopausal women: 20	65.63 ± 5.58 42.75 ± 7.25	220.38 ± 43.48 170.68 ± 30.37	61.25 ± 12.74 62.70 ± 14.87	136.30 ± 39.00 89.85 ± 21.07	114.13 ± 39.42 96.45 ± 42.23
Matthews et al. [14]	1989	Cohort	USA	Postmenopausal women: 65 Premenopausal women: 65	47.80 ± 1.60 47.30 ± 1.50	192.66 ± 44.02 180.31 ± 44.02	60.39 ± 22.06 56.90 ± 17.81	114.40 ± 44.06 108.21 ± 44.06	89.38 ± 70.80 76.11 ± 60.18

*TC*, total cholesterol; *HDL-C*, high-density lipoprotein cholesterol; *LDL-C*, low-density lipoprotein cholesterol; *TG*, triglyceride

### Association between menopause and HDL-C

**Fig. 2:**
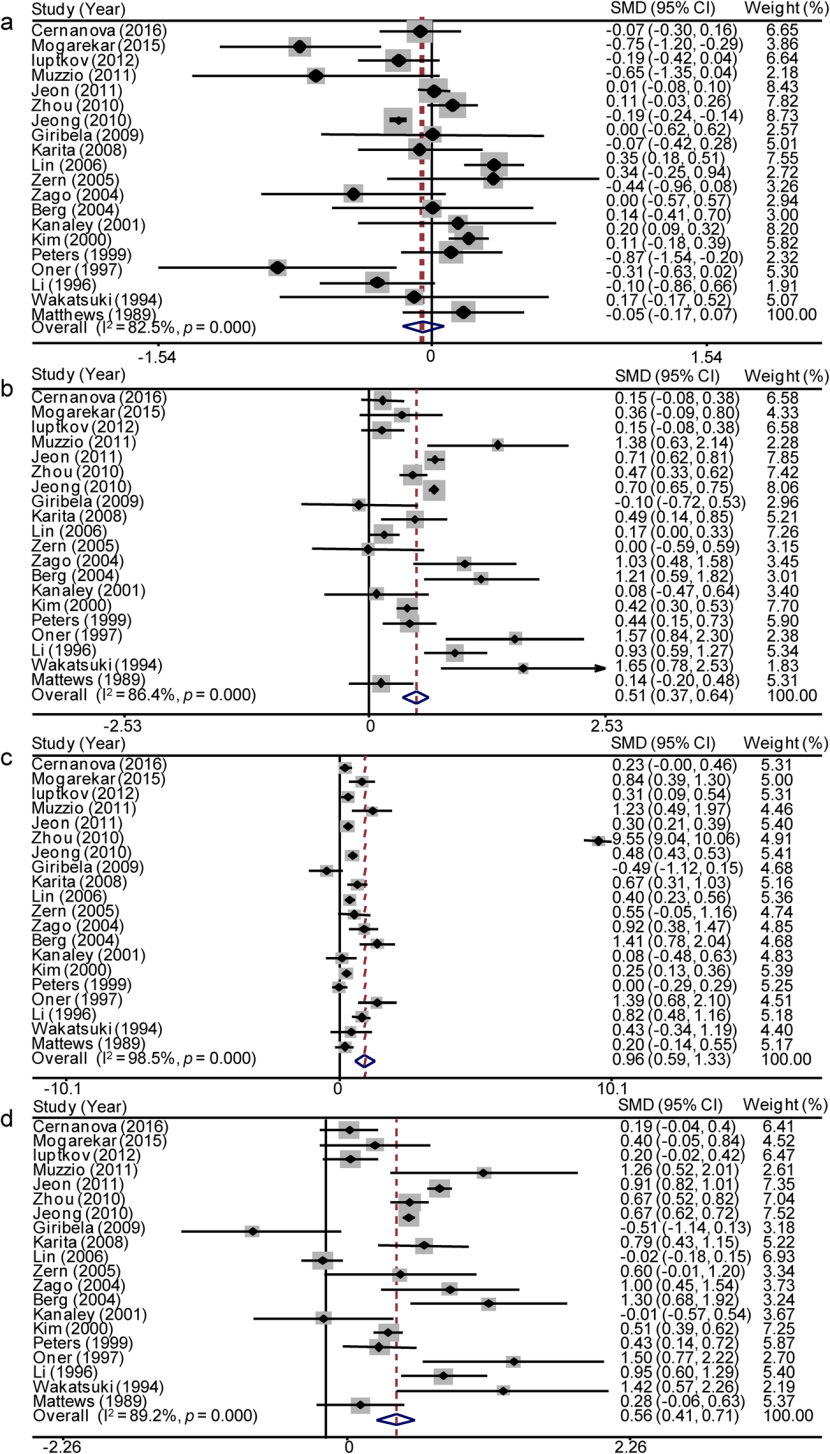
Forest plots of the meta-analysis of HDL-C (a), LDL-C (b), TG (c), and TC (d) levels using random effects models. SMD standardized mean difference, CI confidence interval

**Fig. 3:**
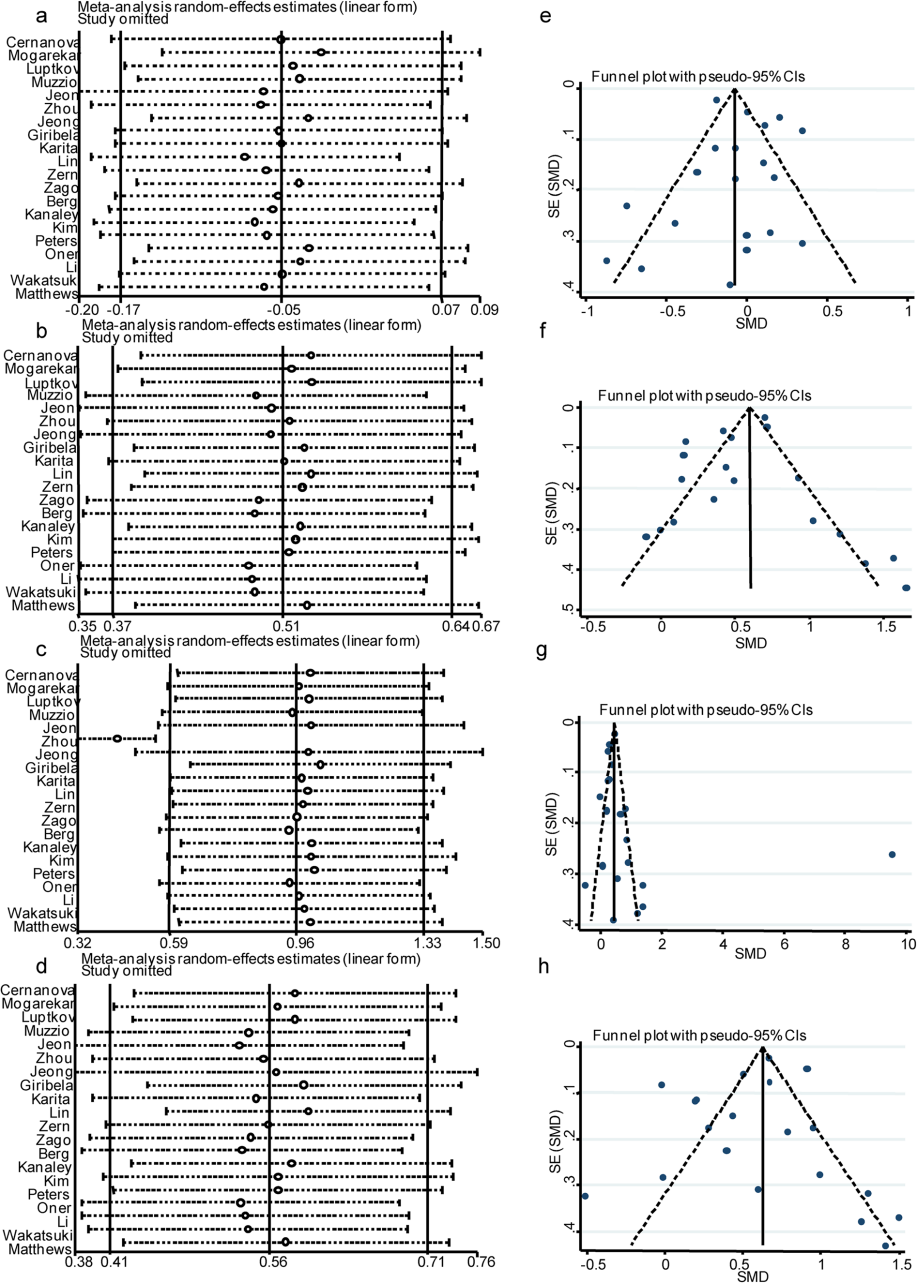
Sensitivity analysis and funnel plot of the meta-analysis of HDL-C, LDL-C, TG, and TC levels. Sensitivity analysis (**a**, **b**, **c**, **d**) and funnel plots (**e**, **f**, **g**, **h**) were performed to examine the heterogeneity of this meta-analysis. SMD standardized mean difference, CI confidence interval

HDL-C levels in the postmenopausal and premenopausal women extracted from the 20 studies were synthesized. Due to heterogeneity, which was > 70%, we conducted a meta-analysis of the 20 included studies using the random effects model to identify differences in HDL-C levels between postmenopausal women and premenopausal women (Fig. 2a). The HDL-C levels were not significantly different between postmenopausal and premenopausal women (SMD = − 0.053, 95% CI − 0.171 to 0.066, *p* = 0.383). Significant heterogeneity was observed among the available studies (*p* < 0.001 and *I*^2^ = 82.5% for the heterogeneity test). A meta-regression analysis was conducted to identify potential moderators of this heterogeneity, but associations between HDL-C levels and covariates, including the publication year, sample size, and country, were not observed (Table 2). We performed a subgroup analysis after stratification by publication year, sample size, and country (Table 3). In the subgroups with a sample size ≤ 500 and non-Asian countries, the *I*^2^ value was significantly reduced by more than 20%. In the sensitivity analysis, no studies which were sequentially omitted affected the association between the menopause status and HDL-C levels (Fig. 3a). A funnel plot was generated and Egger’s test was performed to assess the publication bias of the studies included in this meta-analysis. The shapes of the funnel plots did not reveal any obvious asymmetry and Egger’s test revealed *β* = 0.390 and *p* = 0.616. Both the appearance of the funnel plot and Egger’s test results confirmed a lack of significant publication bias (Fig. 3e).

**Table 2:** The meta-regression analysis of HDL-C, LDL-C, TG, and TC levels

		95% CI		
	*Β*	Lower CI limit	Upper CI limit	*p*
HDL-C				
Publication year	0.094	− 0.123	0.312	0.360
Sample size	0.000	− 0.000	0.000	0.823
Country	0.096	− 0.118	0.311	0.344
Study quality	0.064	− 0.045	0.173	0.235
LDL-C				
Publication year	− 0.014	− 0.045	0.017	0.350
Sample size	0.000	− 0.000	0.000	0.734
Country	− 0.042	− 0.120	0.036	0.276
Study quality	− 0.067	− 0.244	0.109	0.433
TG				
Publication year	0.044	− 0.094	0.183	0.508
Sample size	0.000	− 0.001	0.001	0.951
Country	− 0.187	− 0.519	0.145	0.252
Study quality	1.090	0.454	1.726	0.002*
TC				
Publication year	− 0.013	− 0.045	0.019	0.395
Sample size	0.000	− 0.000	0.000	0.826
Country	− 0.024	− 0.106	0.059	0.558
Study quality	− 0.036	− 0.223	0.152	0.693

The correlation coefficients of publication year, sample size, country, and study quality with 95% confidence intervals (CI) for the effect size of HDL-C, LDL-C, TG, and TC of this meta-analysis are presented and considered significant to be heterogeneity sources with *p* < 0.05(*)

**Table 3:** The subgroup analyses of HDL-C, LDL-C, TG, and TC levels

	*N*	SMD (95% CI) (mg/dl)	*p*	*I*^2^ (%)	Weight (%)
HDL-C					
Total	20	− 0.053 (− 0.171, 0.066)	0.383	82.5	100.00
Sample size					
> 500	5	0.089 (− 0.109, 0.286)	0.379	95.0	40.73
≤ 500	15	− 0.147 (− 0.296, 0.001)	0.052	47.1	59.27
Year					
≥ 2008	9	− 0.119 (− 0.247, 0.010)	0.070	77.8	51.89
< 2008	11	0.036 (− 0.137, 0.210)	0.681	65.4	48.11
Country					
Asia	10	− 0.024 (− 0.190, 0.142)	0.775	90.7	58.91
Not Asia	10	− 0.105 (− 0.242, 0.032)	0.134	20.9	41.09
LDL-C					
Total	20	0.507 (0.373, 0.642)	< 0.001	86.4	100.00
Sample size					
> 500	5	0.506 (0.332, 0.680)	< 0.001	93.3	38.29
≤ 500	15	0.553 (0.323, 0.783)	< 0.001	77.5	61.71
Year					
≥ 2008	9	0.467 (0.304, 0.631)	< 0.001	86.6	51.27
< 2008	11	0.584 (0.353, 0.815)	< 0.001	80.6	48.73
Country					
Asia	10	0.529 (0.373, 0.686)	< 0.001	88.9	57.36
Non-Asian country	10	0.490 (0.215, 0.766)	< 0.001	78.4	42.64
TG					
Total	20	0.958 (0.587, 1.330)	< 0.001	98.5	100.00
Sample size					
> 500	5	2.094 (1.261, 2.926)	< 0.001	99.7	26.47
≤ 500	15	0.529 (0.308, 0.749)	< 0.001	75.7	73.53
Year					
≥ 2008	9	1.436 (0.727, 2.146)	< 0.001	99.4	45.64
< 2008	11	0.500 (0.291, 0.710)	< 0.001	76.2	54.36
Country					
Asia	10	0.467 (0.190, 0.744)	< 0.001	99.3	49.29
Non-Asian country	10	1.416 (0.842, 1.991)	0.001	79.0	50.71
TC					
Total	20	0.563 (0.415, 0.711)	< 0.001	89.2	100.00
Sample size					
> 500	5	0.555 (0.331, 0.779)	< 0.001	96.0	36.09
≤ 500	15	0.592 (0.363, 0.822)	< 0.001	77.5	63.91
Year					
≥ 2008	9	0.535 (0.352, 0.718)	< 0.001	89.5	50.32
< 2008	11	0.634 (0.374, 0.894)	< 0.001	85.2	49.68
Country					
Asia	10	0.610 (0.427, 0.793)	< 0.001	92.1	56.08
Non-Asian country	10	0.511 (0.229, 0.793)	< 0.001	79.6	43.92

*SMD*, standardized mean difference; *CI*, confidential interval

### Association between menopause and the levels of other lipid components

Higher LDL-C (SMD = 0.507, 95% CI = 0.373 to 0.642, *p* < 0.001) (Fig. 2b), TG (SMD = 0.958, 95% CI = 0.587 to 1.330, *p* < 0.001) (Fig. 2c), and TC levels (SMD = 0.563, 95% CI = 0.415 to 0.711, *p* < 0.001) (Fig. 2d) were detected in postmenopausal women than in premenopausal women. No associations between the modifiers publication year, sample size, country, and LDL-C, or TC levels were identified (Table 2) in meta-regression analyses. However, TG levels tended to be affected by study quality (Table 2). The subgroups of a sample size of ≤ 500, year < 2008, and non-Asian countries presented a greater than 20% decrease in the *I*^2^ value. For TC and LDL-C levels, no subgroup showed a significant reduction in the *I*^2^ value (Table 3). The sensitivity analysis revealed a decrease in the random effects estimates after omitting the study conducted by Zhou et al., [5] indicating that this study was a potential source of heterogeneity (Fig. 3c). The funnel plot of the meta-analysis of TG levels showed apparent asymmetry (Fig. 3g), although Egger’s test did not reveal significant publication bias (*β* = 2.779, *p* = 0.281). After removing this study, no association was observed between TG levels and study quality in the meta-regression analysis (*β* = − 0.084, *p* = 0.467), but the synthesized higher TG levels observed in postmenopausal women compared with premenopausal controls were not affected (SMD = 0.433, 95% CI 0.319 to − 0.548, *p* < 0.001). In the sensitivity analysis, no studies which were sequentially omitted affected the association between the menopause status and LDL-C and TC levels (LDL-C: Fig. 3b; TC: Fig. 3d). Both the appearance of the funnel plot (LDL-C: Fig. 3f; TC: Fig. 3h) and Egger’s test results (LDL-C: *β* = − 1.015, *p* = 0.242; TC: *β* = − 0.720, *p* = 0.465) showed no significant publication bias in the meta-analysis of LDL-C and TC levels in postmenopausal and premenopausal women.

## Discussion

To the best of our knowledge, this is the first meta-analysis conducted to identify an association between the menopause status and blood lipid profiles. Based on the results of our study, HDL-C levels in postmenopausal women were not significantly different from those in premenopausal women. This finding is consistent with some large population studies [5, 19], which indicated that menopause did not affect HDL-C levels. In the subgroup analysis, sample size ≤ 500 and non-Asian countries affected the heterogeneity of this meta-analysis. Although the value was not significant, a small sample size potentially affected the association between HDL-C levels and menopause status (Table 3). The difference in HDL-C levels between pre- and postmenopausal women potentially originates from the analysis of cross-sectional data. Wang et al. [32] identified significantly lower HDL-C levels in postmenopausal women than in premenopausal women in longitudinal studies but not in cross-sectional studies. Due to insufficient evidence of HDL-C changes during the menopause transition, more large population-based prospective studies are crucial to determine the association between HDL-C levels and the menopause transition.

Unlike HDL-C levels, LDL-C, TG, and TC levels were significantly increased in postmenopausal women compared with premenopausal controls. A recent large population-based study also observed significantly higher LDL-C and TC levels in postmenopausal women than in premenopausal women, while HDL-C levels were not different between postmenopausal and premenopausal women [33]. Menopause-induced proatherogenic lipid profile changes may mainly affect LDL-C, TG, and TC levels.

The study quality of the included articles was not associated with HDL-C, LDL-C, TG, and TC in meta-regression analyses (Table 2). We strictly followed the inclusion and exclusion criteria to include relevant studies in our meta-analysis. No significant source of heterogeneity was identified and no low-quality studies were included. The sensitivity analysis identified the study conducted by Zhou et al. [5] as a source of heterogeneity in the pooled analysis of TG levels (Fig. 3c), but its omission did not affect the conclusion of increased TG levels in postmenopausal women compared with premenopausal women.

Besides quantitative HDL-C levels, novel metrics for evaluating HDL quality strongly pointed to these levels being independent cardiovascular risk factors. Compared with HDL levels of premenopausal women, less oxidative resistance of HDL among postmenopausal women was demonstrated, together with an impaired ability to inhibit LDL oxidation [25]. On the other hand, large HDL particle concentrations and HDL-mediated cholesterol efflux capacity has been reported to be increased in the early menopausal transition [34]. These novel HDL metrics may explain the pathophysiological changes in late perimenopausal and postmenopausal women, which needs to be confirmed by acquiring additional evidence and performing further studies.

Estrogen, which is the most important female sex hormone, displays decreased levels in postmenopausal women. Hormone replacement therapy and estrogen receptor modulators have been reported to improve lipoprotein levels in postmenopausal populations [35, 36]. Additional studies are needed to explore the effects of sex hormones on postmenopausal lipid functions.

### Strengths and limitations

Our study is, as far as we know, the first meta-analysis to compare the association of HDL-C levels between healthy postmenopausal and premenopausal women, which increases the knowledge base regarding the potential association between HDL-C levels and the menopause status. One of the limitations of this study is that most of the participants came from Asia because of large sample size–based Korean and Japanese studies; thus, more clinical data derived from further studies from Western countries are required to better represent global populations. The definition of menopause status is based on the time from the last bleeding pattern, regardless of sex hormone levels. Additionally, although some studies have used FSH levels as a criterion to confirm menopause status, consensus on the cut-off values for postmenopausal FSH levels has not as yet been established [22, 28]. Another limitation is the small number of included cohort studies and sample sizes; therefore, further prospective studies would help to improve the inferences of causality.

## Conclusions

Based on the evidence of this meta-analysis, HDL-C levels are not associated with menopause status. Higher LDL-C, TG, and TC levels are observed in postmenopausal women than in premenopausal women. Prospective studies with large sample sizes examining HDL-C levels and HDL functions in women with different menopause statuses are needed.

## Electronic supplementary material

ESM 1 (DOCX 44 kb).

## Data Availability

Not applicable.
