# Supplementary material for: Association between HDL-C levels and menopause: a meta-analysis
Source: Hormones (Athens). 2020 Jun 18;20(1):49–59. doi: 10.1007/s42000-020-00216-8 (PMC7889539; doi:10.1007/s42000-020-00216-8)
Supplement: Supplementary file 1 — (DOCX 44 kb). [file 42000_2020_216_MOESM1_ESM.docx]

**Supplementary Tables**

**Table S1.** Assessment of methodological quality using the Cross-Sectional/Prevalence Study Quality tool.

1. Eleven items of the quality assessment recommended by the AHRQ.

| **Item** |
| --- |
| Cross-sectional/ prevalence study quality forms |
| 1. Define the source of information (survey, record review) |
| 2.List inclusion and exclusion criteria for exposed and unexposed subjects (cases and controls) or refer to previous publications |
| 3. Indicate time period used for identifying patients |
| 4. Indicate whether subjects were consecutive if not population-based |
| 5. Indicate if evaluators of subjective components of study were masked to other aspects of the status of the participants |
| 6. Describe any assessments undertaken for quality assurance purposes (e.g., test/retest of primary outcome measurements) |
| 7. Explain any patient exclusions from the analysis |
| 8. Describe how confounding was assessed and/or controlled. |
| 9. If applicable, explain how missing data were handled in the analysis |
| 10. Summarize patient response rates and completeness of data collection |
| 11. Clarify what follow-up, if any, was expected and the percentage of patients for which incomplete data or follow-up was obtained |

1. Total scores for the 13 eligible studies analyzed using the quality assessment recommended by the AHRQ.

| References | Item 1 | Item 2 | Item 3 | Item 4 | Item 5 | Item 6 | Item 7 | Item 8 | Item 9 | Item 10 | Item 11 | Total score |
| --- | --- | --- | --- | --- | --- | --- | --- | --- | --- | --- | --- | --- |
| Cross-sectional/prevalence study quality result | | | | | | | | | | | | |
| **Cernanova et al. [7]** | Yes | Yes | No | Yes | No | No | Yes | Yes | No | Yes | No | 6 |
| **Mogarekar and Kulkarni [16]** | Yes | Yes | No | Yes | No | No | No | No | No | Yes | No | 4 |
| **Luptakova et al. [17]** | Yes | Yes | No | Yes | No | No | Yes | Yes | No | Yes | No | 6 |
| **Muzzio et al. [18]** | Yes | Yes | No | Yes | No | No | Yes | Yes | No | Yes | No | 6 |
| **Jeon et al. [19]** | Yes | Yes | No | Yes | No | No | No | Yes | No | Yes | No | 5 |
| **Zhou et al. [5]** | Yes | Yes | Yes | Yes | No | No | Yes | Yes | Yes | Yes | Yes | 9 |
| **Jeong et al. [20]** | Yes | Yes | Yes | Yes | No | No | No | Yes | No | Yes | No | 6 |
| **Giribela et al. [21]** | Yes | Yes | No | Yes | No | No | No | No | No | Yes | No | 4 |
| **Karita et al. [22]** | Yes | Yes | No | Yes | No | No | Yes | Yes | No | Yes | No | 6 |
| **Lin et al. [23]** | Yes | Yes | Yes | Yes | No | No | No | Yes | No | Yes | No | 6 |
| **Zern et al. [24]** | Yes | Yes | No | Yes | Yes | No | No | No | No | Yes | No | 5 |
| **Zago et al. [25]** | Yes | Yes | No | Yes | No | No | Yes | No | No | Yes | No | 5 |
| **Berg et al. [26]** | Yes | Yes | No | Yes | No | No | No | Yes | No | Yes | No | 5 |
| **Kanaley et al. [27]** | Yes | Yes | No | Yes | No | No | No | Yes | No | Yes | No | 5 |
| **Kim et al. [28]** | Yes | Yes | No | Yes | No | No | No | No | No | Yes | No | 4 |
| **Peters et al. [29]** | Yes | Yes | No | Yes | No | No | Yes | Yes | Yes | Yes | No | 7 |
| **Oner et al. [30]** | Yes | Yes | No | Yes | No | No | No | No | No | Yes | No | 4 |
| **Li et al. [4]** | Yes | Yes | No | Yes | No | No | Yes | No | No | Yes | No | 5 |
| **Wakatsuki and Sagara [31]** | Yes | Yes | No | Yes | No | No | No | No | No | Yes | No | 4 |
| **Matthews et al. [14]** | Yes | Yes | Yes | Yes | No | No | No | No | No | Yes | Yes | 6 |
